# Case of Congenital Deficiency of the Sternum

**Published:** 1845-01

**Authors:** 


*Case of the Congenital Deficiency of the Sternum.*—A man, strong and well-built, 5 ft. 3 in. German (about 5 ft. 4 in. English) in height, and 22 years of age, was found, on examination for military service, with a remarkable deficiency of the sternum. In the middle of the chest the motions of the heart could be perceived at a considerable distance. The sternum was entirely wanting, with the exception of a small rudiment in the situation of the ensiform cartilage, so that the heart was only covered in the middle line of the thorax by the common integuments. On applying the hand, the alternate contractions of the auricles and ventricles could be distinctly perceived. The extremities of the ribs on either side, where they ought to have been in contact with the sternum, were found connected in a continuous, apparently cartilaginous line. The young man declared that he had always enjoyed good health, and that he had never suffered from the deficiency pointed out, in his business of musician.—*London Medical Gazette, from Med. Zeitung.*

